# A new species of *Dysanabatium* Bernhauer and additional records of *D. jacobsoni* Bernhauer (Coleoptera, Staphylinidae, Paederinae)

**DOI:** 10.3897/zookeys.409.6969

**Published:** 2014-05-15

**Authors:** Zhong Peng, Volker Assing, Li-Zhen Li, Mei-Jun Zhao

**Affiliations:** 1 Department of Biology, College of Life and Environmental Sciences, Shanghai Normal University, Shanghai, 200234, P. R. China; 2 Gabelsbergerstr. 230163 Hannover, Germany

**Keywords:** Coleoptera, Staphylinidae, *Dysanabatium*, new species, new records, China

## Abstract

*Dysanabatium hainanense* Peng & Li, sp. n. (Hainan: Wuzhi Shan, Diaoluo Shan) is described and illustrated. Additional records of *D. jacobsoni* Bernhauer, 1915 are reported. The habitus, the sexual characters, and the distribution of this enormously variable species are illustrated.

## Introduction

The paederine genus *Dysanabatium* Bernhauer, 1915 is distributed in the Oriental and southeastern Palaearctic region and was previously represented by seven species. Only one species, *Dysanabatium jacobsoni* Bernhauer, 1915, had been reported from China (Yunnan) (Rougemont 1997). According to Rougemont (1997), *Dysanabatium* species inhabit river banks in woodland habitats.

A study of *Dysanabatium* material from southwestern China, Laos, and Vietnam yielded one species new to science and numerous records of *Dysanabatium jacobsoni*.

## Material and methods

The examined material is deposited in the following public and private collections:

NHMB Naturhistorisches Museum Basel, Switzerland (M. Geiser, I. Zürcher)

NMP National Museum of Natural History, Praha, Czech Republic (J. Hájek)

SNUC Insect Collection of Shanghai Normal University, Shanghai, China

cAss Private collection Volker Assing, Hannover, Germany

The morphological studies were conducted using Stemi SV 11 (Zeiss Germany) and Olympus CX31 microscopes, and a Jenalab compound microscope (Carl Zeiss Jena). The images were prepared using Nikon Coolpix 995, Canon EOS 70D (with an MP-E 65 macrolens), and Canon G12 cameras. The map was created using MapCreator 2.0 (primap) software.

The following abbreviations are used in the text, with all measurements in millimeters:

Body length (BL) length of body from the anterior margin of the mandibles (in resting position) to the abdominal apex.

Forebody length (FL) length of forebody from the anterior margin of the mandibles to the posterior margin of elytra at suture.

Head length (HL) length of head from the anterior margin of the frons to the posterior margin of the head.

Head width (HW) maximum width of head (including eyes).

Antenna length (AnL) length of antenna from the basis of the antenna to the apex.

Pronotum length (PL) length of pronotum along midline.

Pronotum width (PW) maximum width of pronotum.

Elytral length (EL) at the suture from the apex of the scutellum to the posterior margin of the elytra (at the sutural angles).

Aedeagus length (AL) length of the aedeagus from the apex of the ventral process to the base of the aedeagal capsule.

## Species description and additional records

### Dysanabatium hainanense

Peng & Li sp. n.

http://zoobank.org/951C1265-22A1-4186-8D42-FF274643C0AF

http://species-id.net/wiki/Dysanabatium_hainanense

Figs 1A , 2 , 5

#### Type material.

(8 ♂♂, 1 ♀). Holotype: ♂, labelled ‘China: Hainan Prov., 24 km NE Wuzhishan, Wuzhi Shan Guanshandian, 18°53'N, 109°40'E, alt. 650 m 19.iv.2012, Peng & Dai leg.’ (SNUC). Paratypes: 1 ♀, same label data as holotype (SNUC); 4 ♂♂, same data, but ‘18°54'N, 109°41'E, 21.iv.2012, alt. 650–700 m’ (SNUC); 2 ♂♂, same data, but ‘alt. 700 m 18.iv.2012’ (SNUC); 1 ♂, same data, but ‘Lingshui County, Diaoluo Shan, 18°43'N, 109°53'E, 25.iv.2012, alt. 950–1,000 m’ (SNUC).

#### Description.

Measurements (in mm) and ratios: BL 5.89–6.43, FL 3.34–3.45, HL 0.80–0.84, HW 1.05–1.15, AnL 1.94–2.08, PL 0.93–0.98, PW 0.78–0.83, EL 1.11–1.19, AL 0.89–0.93, HL/HW 0.73–0.76, HW/PW 1.35–1.39, HL/PL 0.85–0.86, PL/PW 1.16–1.19, EL/PL 1.19–1.23, diameter of eye: 0.50–0.54.

**Figure 1.**
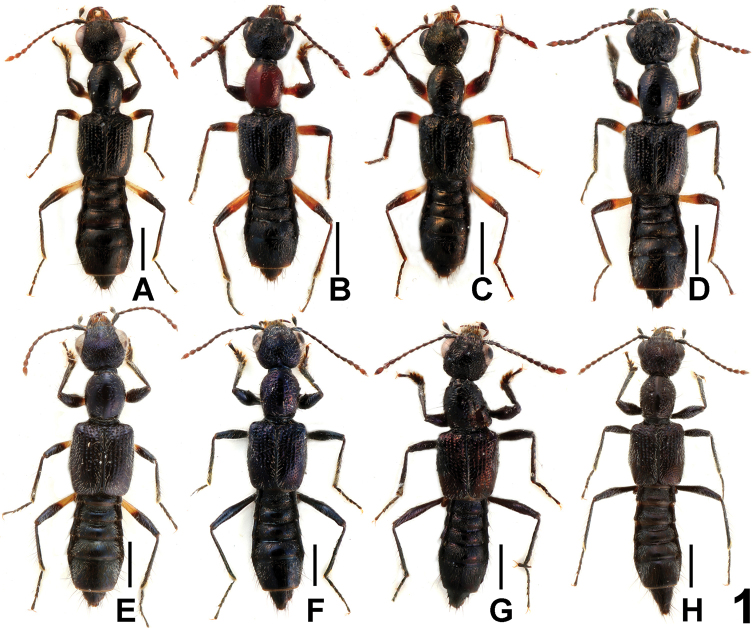
Habitus of *Dysanabatium* spp., **A** *Dysanabatium hainanense* **B–H** *Dysanabatium jacobsoni* (**B–D** Yunnan; **E** Guangxi; **F–H** Hainan). Scales: 1.0 mm.

Habitus as in Fig. 1A. Body entirely black, glossy, devoid of microsculpture; labial palpi and segments I, II, IV of maxillary palpi brownish yellow, segment III of maxillary palpi infuscate; antennae blackish brown at base, gradually becoming paler towards the reddish brown apices; basal halves of femora brownish yellow, distal halves gradually infuscate; tibia and tarsi infuscate.

Head transverse, eyes very large and prominent, temples convergent posteriorly in almost straight line, posterior angles inconspicuous; punctation moderately coarse and dense, sparser in median dorsal portion. All antennomeres oblong.

Pronotum with strongly convex lateral margins in dorsal view; punctation shallow, sparser than that of head; impunctate midline broad.

Elytra with coarse simple punctation arranged in longitudinal series in anterior seven tenths, becoming finer and shallower posteriorly; pubescence golden, erect, conspicuous. Hind wings fully developed.

Abdomen strongly dilated from segments III to apex of segment VI, segment VII convexly tapering posteriorly; segments III–VII with strongly reflexed paratergites; punctation sparse and fine; pubescence fine and pale, with interspersed longer, darker pubescence, especially posteriorly.

Protarsi very strongly, slightly asymmetrically dilated in both sexes; profemora with bases slender, very strongly, symmetrically incrassate to apical third; outer surfaces of tibiae with pale pubescence. First metatarsomere longer than second, subequal to or slightly shorter than fifth; fourth tarsomere simple.

**Figure 2.**
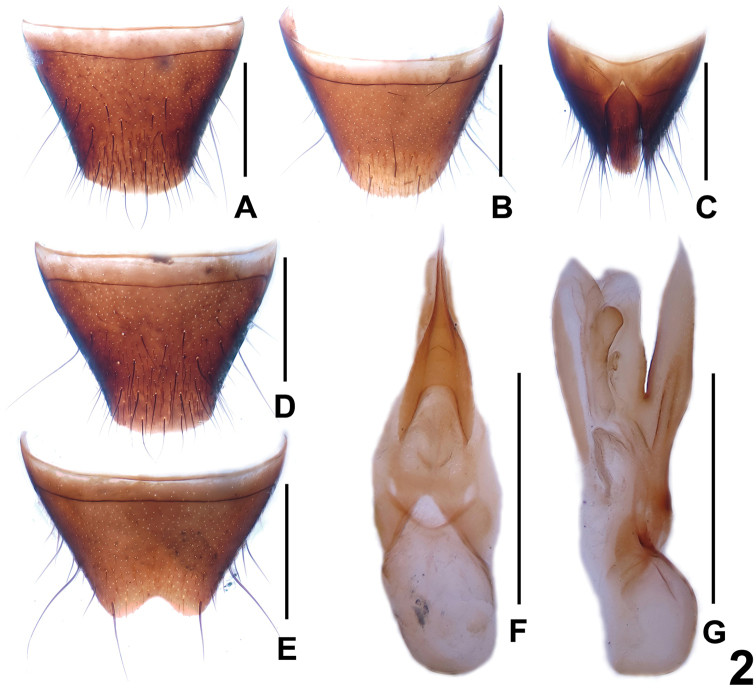
*Dysanabatium hainanense*. **A** female tergite VIII **B** female sternite VIII **C** female tergites IX–X. **D** male tergite VIII **E** male sternite VIII **F** aedeagus in ventral view **G** aedeagus in lateral view. Scales: 0.5 mm.

Male. Posterior margin of tergite VIII (Fig. 2D) broadly convex; sternites III–VI unmodified; posterior margin of sternite VII broadly concave; sternite VIII (Fig. 2E) symmetric and strongly tapering posteriorly, with moderately deep, subtriangular posterior emargination; aedeagus as in Figs 2F, G, ventral process moderately stout and apically acute; internal sac with simple membranous structures and an apically rounded, weakly sclerotized structure.

Female. Posterior margin of tergite VIII (Fig. 2A) asymmetrically and broadly convex; sternite VIII (Fig. 2B) longer than that of male, posterior margin broadly convex; tergite IX (Fig. 2C) with short antero-median portion and moderately long postero-lateral processes; tergite × 5.0 times as long as antero-median portion of tergite IX (Fig. 2C).

#### Distribution and natural history.

The type locality is situated in the Wuzhi Shan and Diaoluo Shan, central Hainan. The specimens from Wuzhi Shan were sifted from flood debris, moss, leaf litter and soil near streams in evergreen broad leaved forest, partly together with *Dysanabatium jacobsoni* Bernhauer, 1915 (Fig. 5).

#### Etymology.

The specific epithet is derived from Hainan, the province where the type locality is situated.

#### Comparative notes.

**Figure 3.**
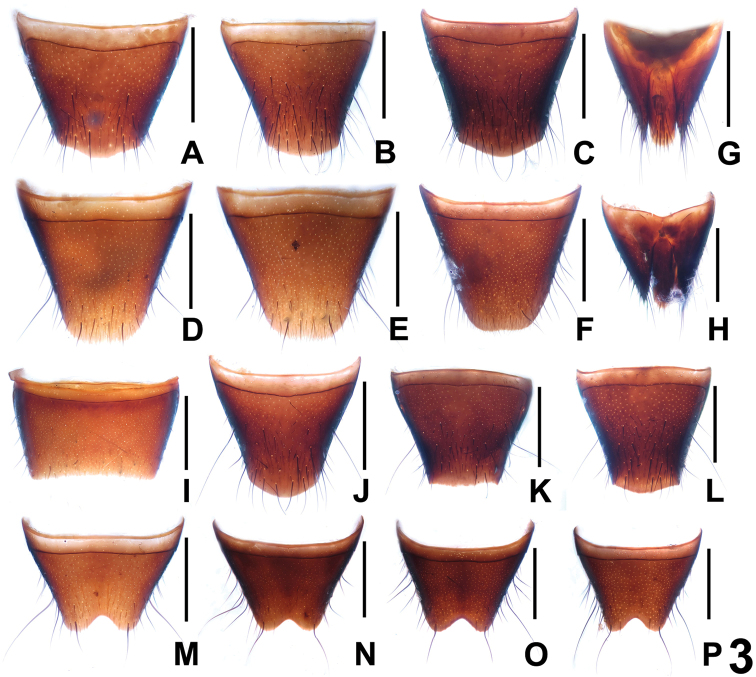
Tergites and sternites of *Dysanabatium jacobsoni*. **A–C** female tergite VIII **D–F** female sternite VIII **G–H** female tergites IX–X. **I** male sternite VII **J–L** male tergite VIII **M–P** male sternite VIII. Scales: 0.5 mm.

**Figure 4. F4:**
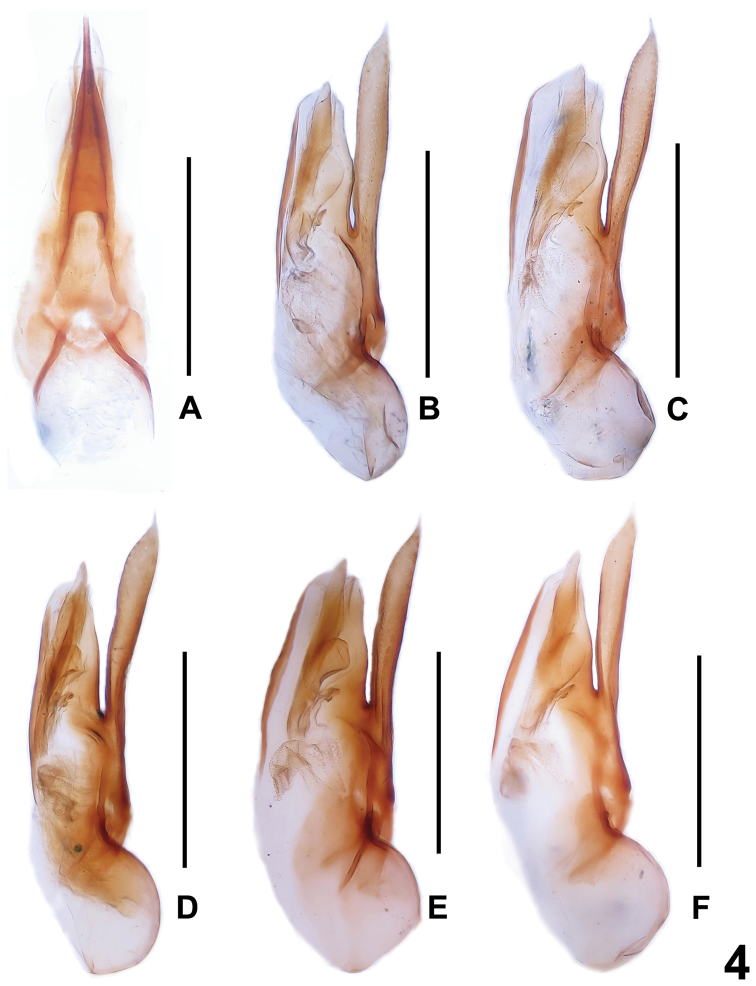
Aedeagus of *Dysanabatium jacobsoni*. **A** aedeagus in ventral view **B–F** aedeagus in lateral view (**A–C** Yunnan; **D** Guangxi; **E–F** Hainan). Scales: 0.5 mm.

**Figure 5. F5:**
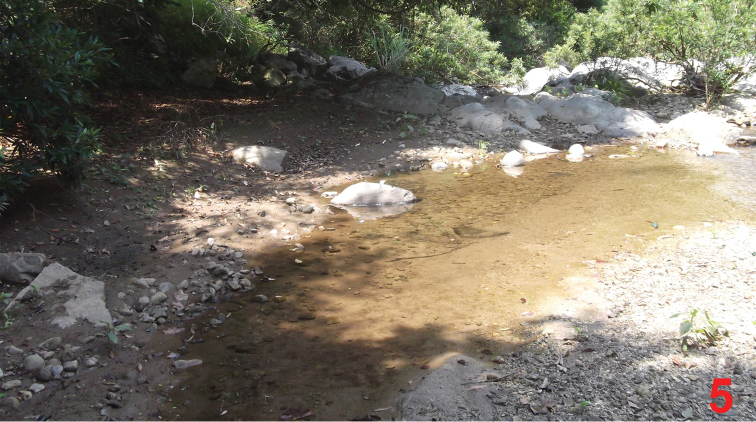
Habitat of *Dysanabatium hainanense* and *Dysanabatium jacobsoni* in the Wuzhi Shan, Hainan.

In external (transverse head; elongate elytra; bicolorous femora) and the male sexual characters (shapes and chaetotaxy of the male sternites VIII), *Dysanabatium hainanense* resembles *Dysanabatium jacobsoni* Bernhauer, 1915 and *Dysanabatium birmana* Cameron, 1931. The new species is distinguished from the former by less prominent posterior angles of the head, somewhat larger eyes, the somewhat shorter fifth metatarsomere, and the morphology of the aedeagus (different shape of ventral process and stouter sclerotized structure in internal sac). For illustrations of *Dysanabatium jacobsoni* see Figs 1B–H, 3A–P, 4A–F and Rougemont (1997: 323, Fig. 1; 324, Figs 3a–5). It differs from *Dysanabatium birmana* by greater body size, the transverse head, the larger eyes, the darker and much longer antennae, the black and glossy pronotum, the denser punctation of the elytra, the larger aedeagus, the somewhat stouter ventral process in lateral view and the weakly sclerotized internal sac of the aedeagus. For illustrations *Dysanabatium birmana* see Rougemont (1997: 324, Fig. 6).

### Dysanabatium jacobsoni

Bernhauer, 1915

http://species-id.net/wiki/Dysanabatium_jacobsoni

**Figure 6.**
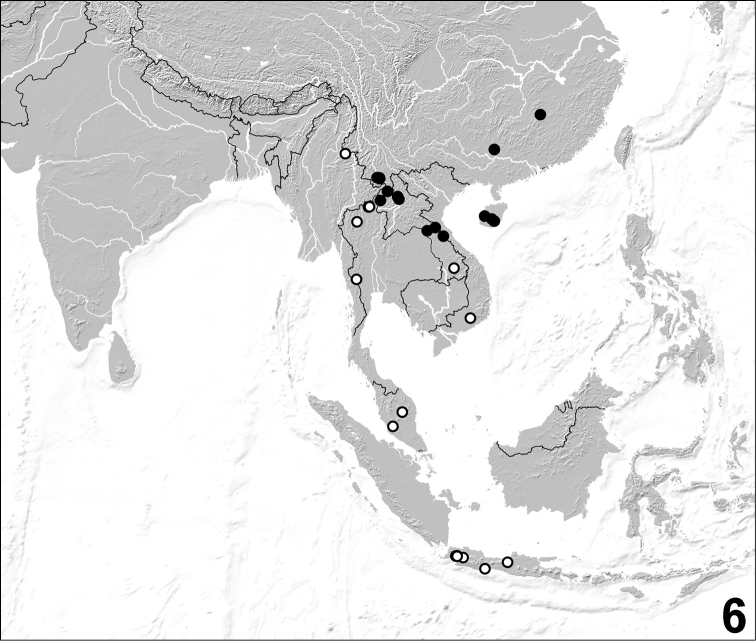
Distribution of *Dysanabatium jacobsoni* in the Oriental and southern East Palaearctic regions (filled circles: examined records; open circles: records from Rougemont (1997)).

Figs 1B–H , 3 [Fig F4] [Fig F5] –6

#### Material studied

(89 ♂♂, 104 ♀♀, 358 exx.)**.** **China:** **Yunnan:** 40 ♂♂, 78 ♀♀, Nabanhe County, Naban, 22°10'N, 100°40'E, 650 m, 7.i.2004, Li & Tang leg. (SNUC); 10 ♂♂, 8 ♀♀, Nabanhe County, Nabanhe N. R., Manfei, 22°09'N, 100°41'E, 650 m, 9.i.2004, Li & Tang leg. (SNUC); 1 ♂, Nabanhe County, Nabanhe N. R., 620 m, 18.ix.2008, Hu & Tang leg. (SNUC); 4 ♂♂, 3 ♀♀, Nabanhe County, Nabanhe N. R., 22°10'N, 100°39'E, 720 m, 22.ix.2008, Hu & Tang leg. (SNUC); 25 ♂♂, 9 ♀♀, Nabanhe County, Mandian, 22°07'N, 100°41'E, 700 m, 12.i.2004, Li & Tang leg. (SNUC); 3 ♀♀, Xishuangbanna, Xiaonuoyouxiazhai, 22°12'N, 100°28'E, 800 m, 6.i.2004, Li & Tang leg. (SNUC). **Guangxi:** 1 ♂, Jinxiu County, Dayaoshan N. R., 24°08'N, 110°11'E, 850–900 m, 24.vii.2011, Peng leg. (SNUC). **Jiangxi:** 1 ♂, Jinggang Shan, Xiangzhou env., 26°36'N, 114°16'E, 370 m, forested stream valley, 26.iv.2011, Fikáček & Hájek leg. (NMP). **Hainan:** 4 ♂♂, 2 ♀♀, 24 km NE Wuzhishan, Wuzhi Shan Guanshandian, 18°53'N, 109°40'E, 650 m 19.iv.2012, Peng & Dai leg. (SNUC); 2 ♂♂, Changjiang County, Bawangling, 19°07'N, 109°07'E, 450–650 m, 13.iv.2010, Zhu leg. (SNUC); 1 ♂, Lingshui County, Diaoluo Shan, 18°43'N, 109°52'E, 900 m, 18.xi.2006, Li leg. (SNUC); 1 ♀, Lingshui County, Diaoluo Shan, 18°43'N, 109°51'E, 1000 m, 20.iv.2010, Yuan leg. (SNUC).

**Vietnam:** 320 exx., Quang Binh province, Vietnam-Laos border region, 1 km N Cha Lo, 17°41'N, 105°46'E, 11.–24.iv.2010, Dembický leg. (NHMB, cAss).

**Laos:** 9 exx., Khammouan province, Ban Khoun Ngeun, 18°07'N, 104°29'E, 200 m, 24.–29.iv.2001, Kubáň leg. (NHMB, cAss); 4 exx., Bokeo province, 5 km W Ban Toup, Bokeo Nature Reserve, 20°27–28'N, 100°45'E, 500–700 m, 4.–18.v.2011, Brancucci et al. leg. (NHMB, cAss); 13 exx., Louangphrabang province, 5 km W Ban Song Cha, 20°33–34'N, 102°14'E, 1200 m, 1.–16.v.1999, Kubáň leg. (NHMB, cAss); 1 ex., Louangphrabang province, Thong Khan, 20°33–34'N, 102°14'E, 750 m, 11.–21.v.2002, Kubáň leg. (NHMB); 6 exx., Oudomxai province, 17 km ENE Oudom Xai, 20°45'N, 102°09'E, 1100 m, 1.–9.v.2002, Kubáň leg. (NHMB, cAss); 3 exx., Bolikhamxai province, 8 km NE Ban Nape, 18°21'N, 105°08'E, 600 m, 1.–18.v.2001, Kubáň leg. (NHMB, cAss); 1 ex., Louangnamtha province, between Namtha and Muang Sing, 21°09'N, 101°19'E, 900–1200 m, 5.–31.v.1997, Kubáň leg. (NHMB); 1 ex., Champasak province, Bolaven plateau, Muang Paxong, Ban Thongvay, 15°14'N, 106°32’, 1000–1200 m, 7.–16.vi.2008, Solodovnikov & Pedersen leg. (cAss).

#### Comment.

The original description of *Dysanabatium jacobsoni* is based on an unspecified number of syntypes from Java (Bernhauer 1915). Two syntypes, a female without head and pronotum labelled as “Type” and a male labelled as “Cotype” from the Bernhauer collection were studied by Rougemont (1997). For illustrations of the habitus and the male and female sexual characters see Figs 1B–H, 3A–P, and 4.

As was already observed by Rougemont (1997), this species is subject to enormous intraspecific variation of external characters (Figs 1B–H) such as body size, coloration, punctation, the shapes of the head and pronotum. The sexual characters are somewhat variable, too, especially the shapes of the posterior margin of tergite VIII (Figs 3A–C, 3J–K) and sternite VIII (Figs 3D–F, 3M–P) in both sexes, and the shapes of the ventral process and the membranous structures of the aedeagus (Figs 4B–F). Specimens from Yunnan are usually relatively small (BL 4.83–6.04 mm, FL 2.95–3.45 mm), have blackish elytra (Figs 1C–D) sharply bicolorous femora, and partly (16 males and 9 females) a bright red pronotum (Fig. 1B). In the specimen from Guangxi the forebody has a faint blueish hue (Fig. 1E) and the sclerotized spine in the internal sac of the aedeagus is slender (Fig. 4D). All the specimens from Hainan have an on average larger body (BL 5.84–6.95 mm, FL 3.37–3.61 mm), a forebody with a weak or distinct metallic hue (Figs 1F–H), entirely black femora, and conspicuous membranous structures in the internal sac of the aedeagus (Figs 4E–F). In the material from Vietnam and Laos, most of the specimens have a black forebody, some have a more or less distinct blueish or greenish metallic hue, and seven specimens from Vietnam have a reddish pronotum. The legs of all the specimens from Vietnam and Laos are bicoloured, but the extent of the yellowish coloration of the femora is highly variable.

#### Distribution and natural history.

According to Rougemont (1997), *Dysanabatium jacobsoni* had been recorded from Indonesia (Java), Malaysia, Thailand, Vietnam, and one locality in the south of the Chinese province Yunnan. The currently known distribution is mapped in Fig. 6. The above specimens from Laos represent new country records. The altitudes of the examined material and of the material seen by Rougemont (1997) range from 200 to 1200 m. The specimens from China were found in flood debris, moss, leaf litter and soil near streams in evergreen broad leaved forest, partly together with *Dysanabatium hainanense* (Fig. 5).

## Supplementary Material

XML Treatment for Dysanabatium hainanense

XML Treatment for Dysanabatium jacobsoni
